# Ppm1d truncating mutations promote the development of genotoxic stress-induced AML

**DOI:** 10.1038/s41375-023-02030-8

**Published:** 2023-09-14

**Authors:** Monika Burocziova, Petr Danek, Anna Oravetzova, Zuzana Chalupova, Meritxell Alberich-Jorda, Libor Macurek

**Affiliations:** 1 Department Cancer Cell Biology, Institute of Molecular Genetics of the Czech Academy of Sciences, Videnska 1083, 142 20 Prague 4, Prague, Czech Republic; 2 Department of Hemato-oncology, Institute of Molecular Genetics of the Czech Academy of Sciences, Videnska 1083, 142 20 Prague 4, Prague, Czech Republic; 3 Childhood Leukaemia Investigation Prague, Department of Pediatric Haematology and Oncology, 2nd Faculty of Medicine, Charles University in Prague, University Hospital Motol, V Uvalu 84, Praha, 150 06 Czech Republic

**Keywords:** Acute myeloid leukaemia, Risk factors, Stem-cell research

## Abstract

Hematopoietic stem cells (HSCs) ensure blood cell production during the life-time of an organism, and to do so they need to balance self-renewal, proliferation, differentiation, and migration in a steady state as well as in response to stress or injury. Importantly, aberrant proliferation of HSCs leads to hematological malignancies, and thus, tight regulation by various tumor suppressor pathways, including p53, is essential. Protein phosphatase magnesium-dependent 1 delta (PPM1D) is a negative regulator of p53 and promotes cell survival upon induction of genotoxic stress. Truncating mutations in the last exon of *PPM1D* lead to the production of a stable, enzymatically active protein and are commonly associated with clonal hematopoiesis. Using a transgenic mouse model, we demonstrate that truncated PPM1D reduces self-renewal of HSCs in basal conditions but promotes the development of aggressive AML after exposure to ionizing radiation. Inhibition of PPM1D suppressed the colony growth of leukemic stem and progenitor cells carrying the truncated PPM1D, and remarkably, it provided protection against irradiation-induced cell growth. Altogether, we demonstrate that truncated PPM1D affects HSC maintenance, disrupts normal hematopoiesis, and that its inhibition could be beneficial in the context of therapy-induced AML.

## Introduction

Hematopoietic stem cells (HSCs) generate all types of hematopoietic progenitor cells and mature blood cells. Self-renewal of HSCs is regulated by various tumor suppressor pathways including p53, ATM, and INK4a/p16 [1–4]. Previous studies showed that p53 affects HSC self-renewal and number [5–7] and that loss of p53 promotes AML [8]. Protein phosphatase magnesium-dependent 1 delta (PPM1D, also known as WIP1 for wild-type p53 induced phosphatase 1) is ubiquitously expressed at basal levels and its expression is stimulated by p53 after various forms of stress including DNA damage [9]. High expression of PPM1D was observed in stem cell compartments in various tissues, including intestinal stem cells [10], neural progenitor cells (NPCs) [11], and hematopoietic stem cells (HSCs) [12]. Forming a negative feedback loop, PPM1D acts as a negative regulator of p53 and protects cells from programmed cell death or senescence thus promoting survival in the presence of DNA damage [13–17]. Besides suppressing p53 response, PPM1D was reported to target other pathways including p38-MAPK and mTORC1 (mammalian target of rapamycin complex 1) [12, 18]. In the context of hematopoiesis, loss of PPM1D impaired the maturation of T cells and the thymic epithelial cells (mTECs) due to sustained activation of p53 and p38-MAPK pathways, respectively [19, 20]. In addition, *Ppm1d* deficient mice showed age-associated phenotypes in HSCs characterized by an enlarged pool of stem cells and a reduced repopulating activity [12]. Interestingly, the expansion of HSC compartment observed in *Ppm1d*^−/−^ mice was mTORC1-dependent, whereas reduction of the repopulating activity was rescued by depletion of p53 [12]. Finally, *Ppm1d*^-/-^ mice exhibited a significant increase in myeloid cells and a decrease in lymphoid cells indicative of defects in the differentiation of HSC and progenitors [12, 21].

Whereas the loss of PPM1D protected mice from the development of cancer in various mouse models [10, 22, 23], PPM1D overexpression or amplification of 17q23 locus carrying the *PPM1D* gene have been reported in various solid tumors, including ~10% of breast cancers [24, 25]. In addition, nonsense or frameshift mutations in exon 6 of *PPM1D* lead to the expression of a C-terminally truncated protein that retains enzymatic activity and shows greatly increased protein stability [26]. We have previously shown that these truncating PPM1D mutations suppress the function of p53 in intestinal stem cells and in T-lymphocytes, impair cell cycle checkpoints, and allow cells to proliferate in the presence of DNA damage [17, 26, 27]. Remarkably, truncating PPM1D mutations are frequently observed in therapy-induced acute myeloid leukemia (t-AML) or myelodysplastic syndrome (t-MDS) patients [28, 29], which confers the ability of HSCs carrying the truncated PPM1D to escape cell death induced by chemotherapy. Further, mosaic mutations in PPM1D have also been identified in the peripheral blood of healthy individuals during aging and have been related to the development of clonal hematopoiesis (CH) [28, 30, 31]. CH arises from a mutated HSC, which gains a fitness advantage and exhibits a disproportionate growth and is now recognized as a risk factor for the development of hematologic malignancies and cardiovascular diseases [32–35]. Large sequencing studies identified PPM1D as one of the drivers of CH [32, 33, 36, 37], however, the pathogenicity of the truncated PPM1D in hematopoiesis still needs to be experimentally validated.

Here, we exploited the previously described mouse model carrying the truncated *Ppm1d*^T^ allele [27] and investigated its oncogenic potential in hematological malignancies. We observed that *Ppm1d*^T/+^ mice show significantly reduced survival and exhibit hematological alterations. Subsequent analysis revealed that while HSCs derived from *Ppm1d*^T/+^ mice are reduced in numbers, their functionality is enhanced. Further, upon exposure to ionizing radiation, hematopoietic stem and progenitor cells (HSPCs) from *Ppm1d*^T/+^ mice showed enhanced growth and were prone to transformation. Accordingly, irradiated *Ppm1d*^T/+^ mice commonly developed acute myeloid leukemia, which demonstrates the pathogenicity of the truncated PPM1D in t-AML patients. Importantly, we found that inhibition of PPM1D, or stimulation of p53 by inhibition of MDM2, suppresses the survival of *Ppm1d*^T/+^ HSPCs and thus could be potentially utilized for preventing the development of therapy-induced myeloid neoplasms in patients carrying *PPM1D* truncating mutations.

## Methods

### Animals and ethical approval

All animal experiments were approved by the local ethical committee (protocol 87/2020). Mice were maintained on a standard diet and 12 h light–dark cycle with free access to food and water. Generation of the *Ppm1d*^T^ mouse strain that carries a frameshift mutation in the exon 6 of *Ppm1d* was described previously [27]. In this study, 10–12- and 58–150-week-old mice were referred to as young and old, respectively. Where indicated, mice were exposed to a sub-lethal (3 Gy) or lethal (6 Gy) dose of the whole body irradiation using Precision X-RAD 225XL equipped with Cu filter (0.5 mm). Where indicated, mice were intraperitoneally injected with 150 mg/kg of 5-fluorouracil in PBS or with PBS every 14 days. Blood counts and weight were assessed every 7 days.

### Flow cytometry

Murine blood samples were obtained by bleeding from the cheek. Blood was analyzed with an Auto Hematology Analyser (Mindray). Mice were sacrificed by cervical dislocation, femurs, and tibias were isolated, and crunched using a pestle and mortar. For flow cytometric analysis, single cell suspensions of peripheral blood (PB) and bone marrow (BM) from WT and Ppm1d^T/+^ mice were subjected to red cell lysis. Cells were stained with fluorescently-labeled antibodies and analyzed using a FACSymphony (BD Biosciences) flow cytometer. Data were obtained using Diva software (BD Biosciences) and analyzed using FlowJo software (Tree Star Incorporation). Sorting of BM HSCs was performed by two subsequent steps. First, the Lin^+^ fraction of the BM cells were labeled using biotinylated lineage markers CD45/B220 (RA3-6B2), CD3 (145-2c11), Ter119 (TER-119), Gr1 (RB6-8C5), and CD11b (M1/70). These cells were then incubated with anti-biotin magnetic beads (Miltenyi Biotec) and were isolated using MACS separator. Second, the Lin^-^ fraction of the BM was labeled with the c-Kit (2B8), Sca-1 APC (E13-161.7), CD48 FITC (HM48-1), CD150 Pe-Cy7 (TC15-12F12.2) antibodies and with streptavidin-eFluor450. Cell suspensions were stained with Hoechst 33258 to exclude dead cells and HSCs were sorted using Influx instrument (BD Biosciences) as Lin^-^ c-Kit^+^ Sca-1^+^ CD48^-^ CD150^+^ cells as described [38].

### Extreme limiting dilution transplantation

C57BL/6NCrl mice (CD45.1^+^) at the age of 10–12 weeks were used as recipients and were lethally irradiated (6 Gy) prior to transplantation. Donor cells were isolated from WT and PPM1D^T/+^ (Ly5.2^+^) murine BM. HSCs, defined as LKS CD48^−^, CD150^+^ were sorted and intravenously transplanted at three different doses (5, 10, and 20 cells) along with 5 × 10^5^ BM cells as support (Ly5.1^+^). BM and blood of recipients were analyzed 16 weeks after transplantation. Cells were stained with anti-Ly5.1 and Ly5.2 antibodies to distinguish donor-derived cells from the support cells, and with lineage-specific antibodies CD11b, Gr1, B220, and CD3 to assess the reconstitution of myeloid cells, B-cells, and T-cells, respectively. A recipient mouse was defined as positive when engraftment of donor cells was ≥0.1% and presented at least two lineages reconstituted. The frequency of HSCs was calculated with ELDA online software using Poisson statistics and the method of maximum likelihood to the proportion of negative recipients in a limiting dilution setting [39].

### RNA sequencing

RNA sequencing and analysis of HSCs (defined as Lin^−^ c-Kit^+^, Sca-1^+^, CD48^−^, CD150^+^ cells) sorted from 12-week-old WT and Ppm1D^T/+^ mice (*n* = 4) using Influx instrument was performed. RNA was extracted with the RNAeasy Micro Kit (Qiagen) and cDNA was synthesized using the SMARTer Stranded Total RNA-Seq Kit v2 Pico (Takara Bio) according to the manufacturer’s instructions. Alternatively, RNA was isolated from c-Kit+ cells isolated from the leukemic animals using KAPA mRNA HyperPrep Kit (Kapa Biosystems). Sequencing was performed by NextSeq 550 system (Illumina) using NextSeq 500/550 High Output Kit v2. Sequences were mapped to GRCm39 reference genome by STAR v2.7.9 software. The raw counts for each transcript were calculated using featureCounts software from Rsubread package for R software [40]. Differential expression and normalized counts were determined by DESeq2 package in R. Genes with expression change higher than log2FoldCHange > 1 and *p*-adjusted value < 0.05 were considered as significantly up- or down-regulated. Gene Set Enrichment Analysis was performed as described [41]. All genes were pre-ranked based on the statistical significance of gene expression change -log10(*p*-adjusted value) for leukemic samples, −log10 (*p*-value) for HSC, and their enrichments in the gene sets available at MSigDB were analyzed. Gene expression patterns in AML patients versus healthy individuals were obtained from TNMplot database [42].

## Results

### Truncated *Ppm1d* impairs normal hematopoietic development and enhances HSC function

**Fig. 1:**
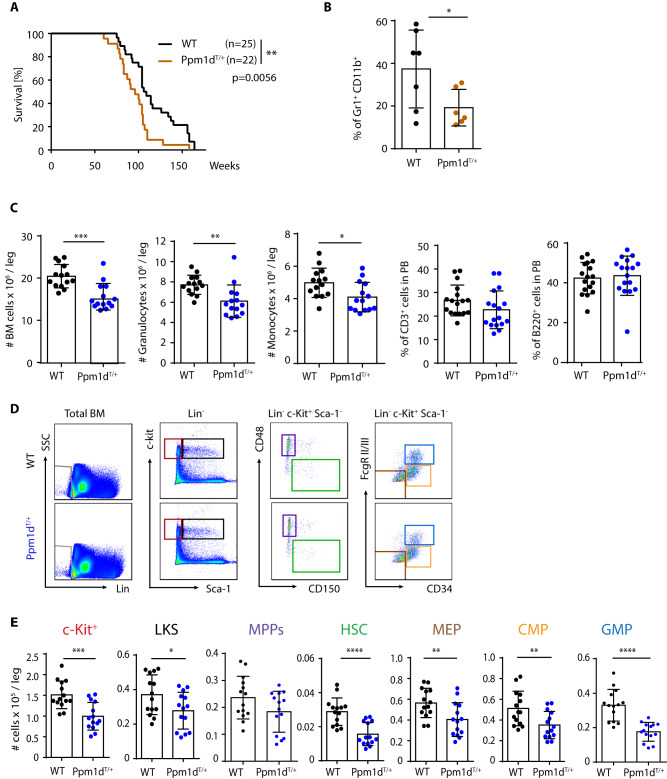
Truncated *Ppm1d* impairs normal hematopoietic development and enhances HSC function. **A** Kaplan–Meier survival analysis of WT (*n* = 25) and *Ppm1d*^*T/+*^ (*n* = 22) mice. Mantle–Cox test was used to assess the statistical significance of obtained results (*p* = 0.0056). **B** Percentage of Gr1^+^ CD11b^+^ cells in PB of 60-week-old mice. Each symbol represents one mouse. At least six mice were used per group. Data represent mean ± s.d. Statistical significance was determined by 2-tailed Student’s *t*-tests (*p < 0.05). **C** Number of BM cells, monocytes (CD11b^+^Ly6C^+^), and granulocytes (CD11b^+^Ly6G^+^) per leg and percentage of CD3^+^ (T cells) and B220^+^ (B cells) in peripheral blood of 10–12-week-old mice. Each symbol represents one mouse. Data represent mean ± s.d. Statistical significance was determined by 2-tailed Student’s *t*-tests (***p < 0.001, **p < 0.01, *p < 0.05). **D** Representative flow cytometry plots from 10–12-week-old WT (upper plots) and *Ppm1d*^*T/*+^ (lower plots) mice. Color boxes indicate the following populations: Lin^−^ cells (gray), Lin^−^ c-Kit^+^ Sca-1^−^ (c-Kit^+^, red), Lin^−^ c-Kit^+^ Sca-1^+^ (LKS, black), Lin^−^ c-Kit^+^ Sca-1^+^ CD48^+^ CD150^−^ (MPPs, violet), Lin^−^ c-Kit^+^ Sca-1^+^ CD48^−^ CD150^+^ (HSCs, green), Lin^−^ c-Kit^+^ Sca-1^+^ CD34^−^ FcgRII/III^−^ (MEP, brown), Lin^−^ c-Kit^+^ Sca-1^+^ CD34^+^ FcgRII/III^low^ (CMP, orange), and Lin^−^ c-Kit^+^ Sca-1^+^ CD34^-^ FcgRII/III^high^ (GMP, blue). **E** Quantification of absolute number of distinct stem and progenitor populations in BM isolated from 10–12-week-old mice. Each symbol represents one mouse. Data represent mean ± s.d. Statistical significance was determined by 2-tailed Student’s *t*-tests (****p < 0.0001, ***p < 0.001, **p < 0.01, *p < 0.05).

Since truncating mutations in *PPM1D* were identified in patients suffering from therapy-related hematological disorders [28, 29], we employed our previously established *Ppm1d*^T/+^ mouse model [27] to investigate the impact of this gain-of-function allele on hematopoiesis (Supplimentary Fig. 1A). Similarly to other tissues, the truncated *Ppm1d*^T^ was present at higher levels than the full length *Ppm1d* in the bone marrow (BM), which is consistent with its previously reported increased protein stability [26, 27] (Suppl. Fig. 1B). *Ppm1d*^T/+^ mice reached adulthood with no signs of disease, however, we observed that *Ppm1d*^T/+^ mice died significantly earlier than the control littermates carrying the wild-type Ppm1d (WT) (Fig. 1A). In addition, analysis of the moribund *Ppm1d*^T/+^ animals indicated reduced percentage of granulocytes in the peripheral blood (PB), suggesting possible hematopoietic alterations caused by the truncated Ppm1d (Fig. 1B). Accordingly, analysis of 60-week-old mice revealed significantly suppressed bone marrow (BM) cellularity, decreased number of HSCs and progenitor populations as well as reduced number of granulocytes and monocytes in BM of *Ppm1d*^T/+^ mice (Supplimentary Fig. 1C, D). Similar BM alterations were already present in young *Ppm1d*^T/+^ mice in comparison to WT littermate controls (Fig. 1C–E and Supplimentary Fig. 1E). Nevertheless, no differences were observed in the lymphoid cell production (Fig. 1C and Supplimentary Fig. 1C). Altogether, these results indicate that the increased activity of the truncated *Ppm1d*^T/+^ protein alters normal hematopoietic development and accelerates mortality of mice.

### Ppm1d^T/+^ HSCs exhibit reduced symmetric self-renewal divisions and enhanced fitness upon transplantation

**Fig. 2:**
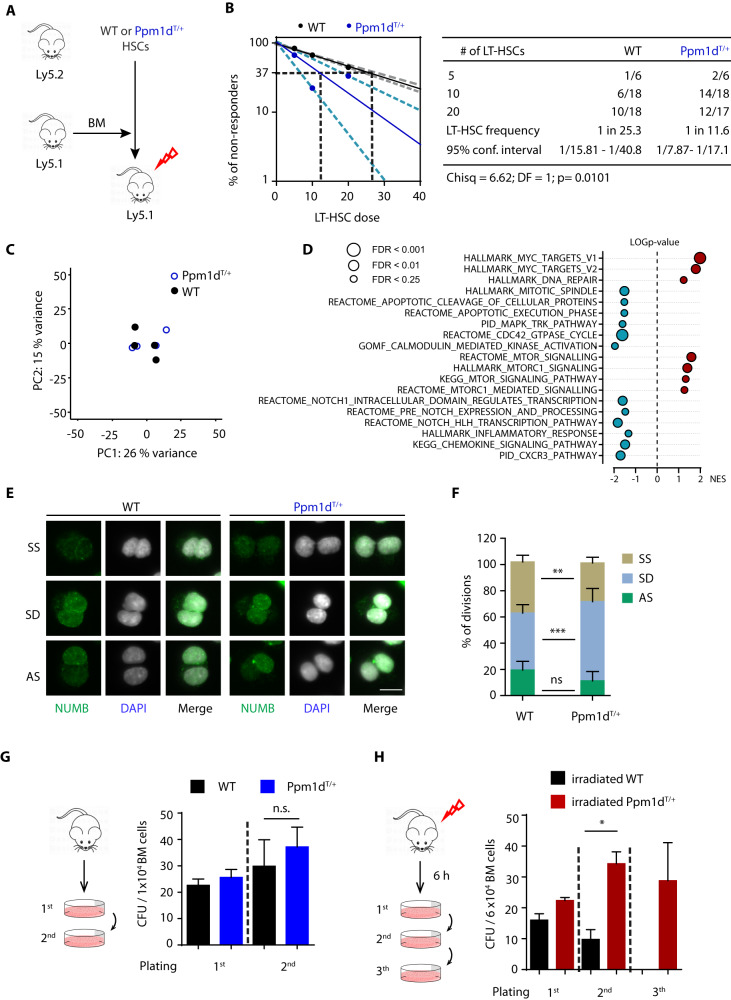
Functional and transcriptomic analysis of *Ppm1d*^T/+^ HSCs. **A** Transplantation scheme of 12-week-old WT and PPM1D^T/+^ HSCs. **B** The frequency of functional HSCs in WT (black) and *Ppm1d*^T/+^ (blue) mice was measured by limiting dilution competitive repopulation unit assays and calculated using ELDA online software based on Poisson distribution statistics (Chi-square test; Chisq = 6.62; *p* = 0.0101). The graph shows the curve fit of the log fraction of nonresponding mice (solid lines) and confidence intervals (dashed lines) versus the number of mice tested. Logarithmic plot; X-axis indicates the dose of transplanted cells and y-axis percentages of negative responders. Reconstitution was evaluated in blood of recipient mice 16 weeks after transplantation. A responder mouse was defined as engraftment ≥0.1% Ly5.2+ cells and contribution ≥0.5% in at least two out of three lineages (T, B, and myeloid cells). **C** Principal component analysis (PCA) of four WT (black symbols) and four *Ppm1d*^T/+^ HSC samples (blue symbols). **D** Gene Set Enrichment Analysis (GSEA) shows the top relevant pathways upregulated or downregulated in *Ppm1d*^T/+^ HSCs compared to WT HSCs. Data were generated using MSigBD Hallmark gene set v.7 (ranked according to NES values, FDR < 0.25). **E** Numb staining (green) in sorted WT and *Ppm1d*^T/+^ HSC after one division. DNA was counterstained with DAPI (white). Representative images of symmetric self-renewal (SS), symmetric differentiation (SD), and asymmetric (AS) division are shown. The scale bar represents 10 µm. **F** Quantification of the cell division types in WT and *Ppm1d*^T/+^ HSCs from E. Symmetric self-renewal (SS), symmetric differentiation (SD), and asymmetric (AS) division. Data represent mean ± s.d. Statistical significance was determined by 2-tailed Student’s *t*-tests (***p < 0.001, **p < 0.01, ns: not significant). At least 50 cells were counted per sample (*n* = 7 WT and *n* = 5 *Ppm1d*^T/+^ mice). **G** Schematic representation of the colony culture replating assays in WT and *Ppm1d*^T/+^ BM cells using MethoCult GF M3434. A total of 1 × 10^4^ BM cells were plated per well. Colonies were counted and replated on day 7. At least three mice were used per group in two separate experiments. Data represent mean ± s.d. Statistical significance was determined by 2-tailed Student’s *t*-tests (n.s. not significant). **H** Schematic representation of the colony culture replating assays. Mice were exposed to 3 Gy sub-lethal IR. Six hours (h) after exposure a total of 6 × 10^4^ BM cells was plated per well. Colonies were counted and replated on day 7. At least three mice were used per group in two separate experiments. Data represent mean ± s.d. Statistical significance determined by 2-tailed Student’s *t*-tests (*p < 0.05).

Given the reduced number of phenotypically defined HSCs in the BM of *Ppm1d*^T/+^ mice, we next investigated whether this phenotypical reduction also affected their functionality. To this end, we performed extremely limiting dilution transplantation assays using 10–12-week-old donors (Fig. 2A). Analysis of PB of the recipient mice 16 weeks post-transplantation showed that the long-term repopulating ability of *Ppm1d*^T/+^ HSCs was improved in comparison to the WT HSCs, while no changes in lineage bias were observed (Fig. 2B and Supplimentary Fig. 2A). To understand how the truncated Ppm1d protein enhances HSCs function, we performed gene expression profiling in HSCs isolated from the WT and *Ppm1d*^T/+^ animals. Nevertheless, consistently with the role of *Ppm1d*^T/+^ as a negative regulator of the stress response, we did not find any statistically significant changes in gene expression under steady state conditions (*p*-adjusted value < 0.05) (Fig. 2C and Supplimentary Fig. 2B). Next, we ranked all transcripts from the most significantly upregulated to the most downregulated in *Ppm1d*^T/+^ HSC based on the logarithmic transformation of their *p* values and performed gene set enrichment analysis (GSEA). We observed that *Ppm1d*^T/+^ HSCs showed tendencies towards deregulation of the pathways related to apoptosis, which correlates with previously reported PPM1D function [17, 29, 43] (Fig. 2D and Supplimentary Fig. 2C). Further, we noted that the transcriptome of *Ppm1d*^T/+^ HSCs shows enrichment of MYC, mTOR, and negative enrichment of NOTCH pathway signatures, which have been previously related to control symmetry of HSC division [44–46] (Fig. 2D and Supplimentary Fig. 2C). This prompted us to investigate the HSC cell division types based on NUMB distribution as previously described [47]. We quantified division types of HSCs doublets treated with cytokinesis inhibitor cytochalasin B, and found a significant increase in cellular differentiation and a decrease of symmetric self-renewal divisions in *Ppm1d*^T/+^ in comparison to WT HSCs (Fig. 2E, F). These results are in agreement with the high activity of mTOR pathway, which leads to HSC differentiation, and with the role of NUMB as a suppressor of Notch signaling pathway [44, 45, 48, 49]. In addition, we noted that the expression level of *Numb* was comparable in *Ppm1d*^T/+^ and WT HSC HSCs (Supplimentary Fig. 2D). In contrast, *Ppm1d*^T/+^ HSC showed a lower expression of Calcium/Calmodulin Dependent Protein Kinase I (*Camk1*) which has been implicated in Numb phosphorylation and is required for asymmetric division [50, 51]. Accordingly, GSEA analysis revealed reduced activation of Calcium/calmodulin-dependent protein kinase pathway in Ppm1d^T/+^ HSCs. (Supplimentary Fig 2D, Fig. 2D). Altogether, our results indicate that despite mild gene expression changes, *Ppm1d*^T/+^ HSCs are more active and inclined to differentiate, thus probably contributing to reducing the HSC pool.

### Enhanced colony forming potential upon genotoxic stress in *Ppm1d*^T/+^ BM cells

To further investigate the functional impact of truncated Ppm1d on HSC function, we performed colony culture replating assays [38]. We found that the colony forming potential of the WT and *Ppm1d*^T/+^ BM cells was comparable, and we observed no significant differences in the steady-state conditions (Fig. 2G and Supplimentary Fig. 2D). However, since mutations in *PPM1D* have previously been implicated in increased cell survival upon exposure to DNA damage-inducing chemotherapy or radiotherapy [17, 27–29], we next investigated the effects of ionizing radiation on the WT and *Ppm1d*^T/+^ BM cells. We observed that the number of colonies was slightly increased in irradiated *Ppm1d*^T/+^ BM cells in comparison to irradiated WT BM cells in the first round of plating (Fig. 2H). Further, while WT cells lost the ability to form colonies in sub-sequent replatings, *Ppm1d*^T/+^ cells formed colonies in secondary and tertiary plating assays, suggesting an enhanced potential to expand during in vitro culture after exposure to irradiation (Fig. 2H and Supplimentary Figs. 2E, 2F). Altogether, these assays indicate that *Ppm1d*^T/+^ BM cells perform better after DNA damage, suggesting that inactivation of p53 signaling pathway by the truncated Ppm1d protein may confer a growth advantage.

### Truncated *Ppm1d* promotes AML after sequential exposure to ionizing radiation

**Fig. 3:**
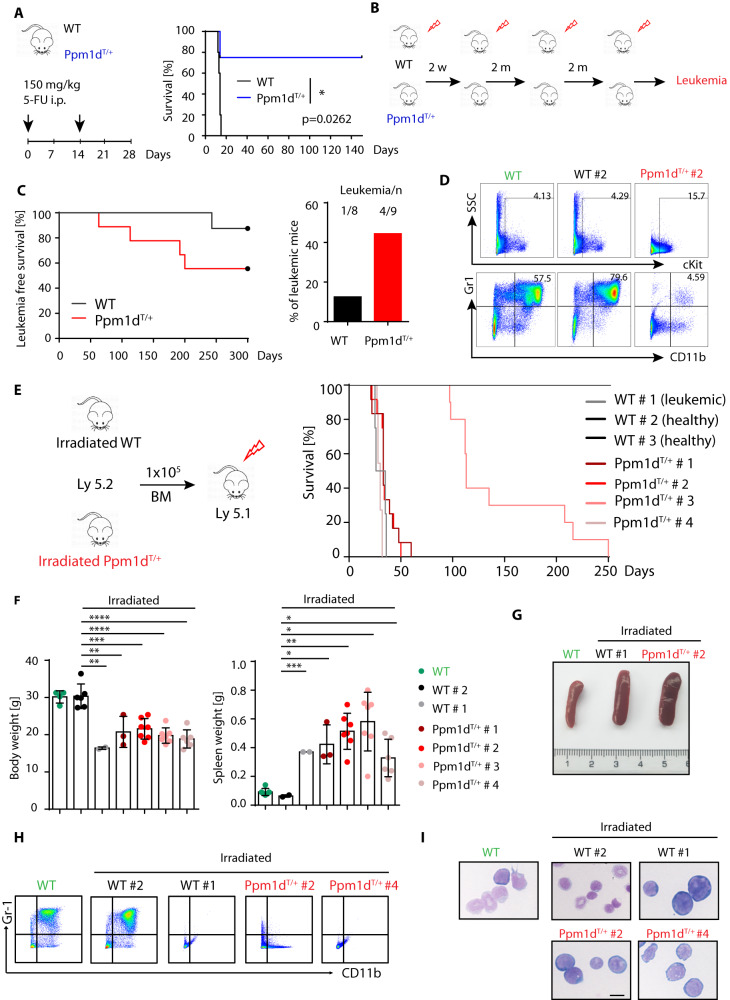
Increased incidence of leukemic transformation in *Ppm1d*^T/+^ mice upon irradiation. **A** Schematic representation of the experimental setup (left panel). WT and *Ppm1d*^*T/+*^ mice were injected intraperitoneally (i.p.) with 150 mg/kg 5-FU as indicated (black arrows). The x-axis indicates days. Kaplan–Meier survival analysis of four WT and four *Ppm1d*^T/+^ mice intravenously injected with 5-FU as indicated in the left panel. Mantle–Cox test was used to assess the statistical significance of obtained results (*p* = 0.0262). **B** Schematic representation of the experimental setup. 12-weeks-old WT and *Ppm1d*^*T/+*^ mice were irradiated with 3 Gy sub-lethal dose and subjected to 4 irradiation cycles. The interval between irradiation is indicated as weeks (w) or months (m). Each group contained at least 8 animals from 2 independent experiments. **C** Leukemia-free survival of mice exposed to sub-lethal irradiation as defined in panel B. Proportion of animals with leukemia in WT and *Ppm1d*^T/+^ mice from Kaplan–Meier survival plot is indicated on the right. **D** Representative flow cytometry plots from indicated mouse genotypes: Healthy non-irradiated mouse (WT; green), healthy irradiated mouse (WT#2; black), and leukemic *Ppm1d*^T/+^ mouse (*Ppm1d*^T/+^#2; red). Numbers indicate the percentage of cKit^+^ cells (upper box) and Gr1^+^CD11b^+^ granulocytes (lower box) in total BM. **E** Re-transplantation scheme of WT and *Ppm1d*^T/+^ primary leukemias from panel C. 1 × 10^5^ primary leukemic WT and *Ppm1d*^T/+^ (Ly 5.2) BM cells were transplanted into sub-lethally irradiated recipient mice (Ly5.1). Leukemia-free survival of re-transplanted leukemic mice is shown. At least 8 mice were used per group. **F** Body weight (left) and spleen weight (right) of non-treated healthy WT mice (green), and mice re-transplanted with BM cells from irradiated donors. The weight of the body and spleen is presented as mean ± s.d. Statistical significance was determined by using 2-tailed Student’s *t*-tests (***p < 0.001, **p < 0.01, *p < 0.05). At least three mice were used per group. **G** Representative pictures from spleens isolated from a healthy WT mouse (WT), a WT irradiated leukemic mouse (WT#1), and a *Ppm1d*^T/+^ irradiated leukemic mouse (Ppm1d^T/+^#2). **H** Representative flow cytometry plots from indicated mouse genotypes: healthy WT control (WT), irradiated WT non-leukemic (WT#2), irradiated WT leukemic (WT#1), irradiated *Ppm1d*^T/+^ leukemic (Ppm1d^T/+^#2), and irradiated *Ppm1d*^T/+^ leukemic (Ppm1d^T/+^#4). Plots show Gr1 CD11b expression in BM. The upper right quadrant shows granulocytes (Gr1^+^CD11b^+^). **I** BM cytospins stained with May-Grünwald Giemsa. Representative images are shown: healthy WT control (WT), irradiated WT non-leukemic (WT#2), irradiated WT leukemic (WT#1), irradiated *Ppm1d*^T/+^ leukemic (Ppm1d^T/+^#2), and irradiated *Ppm1d*^T/+^ leukemic (Ppm1d^T/+^#4). The scale bar represents 10 µm.

We have previously reported that the *Ppm1d*^T^ allele protects mice from the acute cytotoxic effects of a single high dose of ionizing radiation [17]. Interestingly, *Ppm1d*^T/+^ mice surviving this initial insult showed rare cases of thymic lymphoma but the majority of mice did not develop tumors [17]. As truncating PPM1D mutations frequently occur in therapy-induced AML and MDS [28, 29], we decided to explore the connection between truncating PPM1D mutations, exposure to genotoxic agents, and cancer development. In line with our previous observations, we found that *Ppm1d*^T/+^ mice showed improved survival upon acute administration of 5-fluorouracil (5-FU), a commonly used chemotherapeutic genotoxic drug that also induces BM ablation (Fig. 3A). Surprisingly, *Ppm1d*^T/+^ mice that recovered from a high dose of 5-FU survived to adulthood without signs of cancer development, suggesting that a single DNA damaging insult may not be sufficient to complete cellular transformation in *Ppm1d*^T/+^ mice. Nevertheless, we hypothesized that sequential induction of several cycles of DNA damage may better mimic the situation during cancer therapy and may eventually lead to the growth of tumors and/or the development of hematological disorders. Therefore, we subjected WT and *Ppm1d*^T/+^ mice to four subsequent rounds of mild doses of irradiation (3 Gy) and monitored them by flow cytometry for signs of hematological disease (Fig. 3B and Suppl. Fig. 3A). Interestingly, we observed that *Ppm1d*^T/+^ mice commonly developed leukemia within 2–7 months after irradiation and exhibited reduced leukemia free survival in comparison to WT littermates (Fig. 3C, D). In particular, while 4 out of 9 *Ppm1d*^T/+^ mice (44.4%) developed leukemia after sequential irradiation, only 1 out of 8 WT mice (12.5%) did. Next, we transplanted the BM cells from the irradiated animals to WT recipient mice and observed decreased survival of the recipient animals confirming the development of aggressive leukemias in one WT (WT #1) and four *Ppm1d*^T/+^ animals (Ppm1d^T/+^ #1–4) (Fig. 3E). BM transplantation of the other 2 WT donors (WT #2 and 3), which did not developed leukemia, did not affect the survival of recipient mice (Fig. 3E). On the contrary, mice transplanted with *Ppm1d*^T/+^ primary leukemic cells (Ppm1d^T/+^ #1–4) demonstrated a significant loss of the body weight, and typical symptoms of leukemia, including splenomegaly, lack of mature myeloid cells and presence of immature blast cells in the BM (Fig. 3F–I). Altogether, these experiments demonstrate that *Ppm1d*^T/+^ mice are prone to develop AML upon repeated genotoxic insults.

### *Ppm1d*^T/+^ irradiation-induced AML in mice share characteristics with human AML

**Fig. 4:**
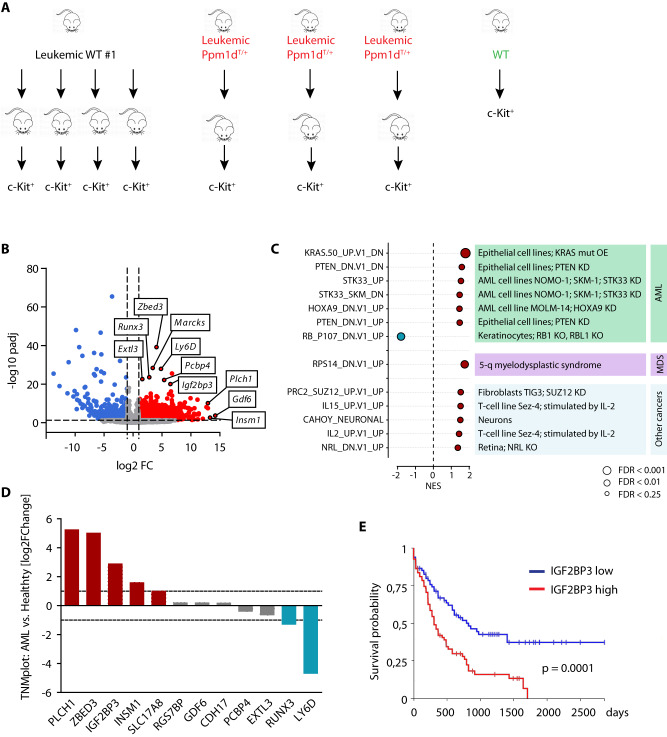
Gene expression profiling of *Ppm1d*^*T/+*^ irradiation-induced AML. **A** Schematic representation of the experimental design. 1 × 10^5^ of primary leukemic cells from WT #1 and *Ppm1d*^*T/+*^#2,3,4 mice were re-transplanted into sub-lethally irradiated recipient mice (Ly5.1). c-kit^+^ cells were sorted for RNA sequencing. **B** Volcano plot showing differentially expressed genes, upregulated (red, log2FC > 1, *p*-adjusted value < 0.05) or downregulated (blue, log2FC < (−1), *p*-adjusted value < 0.05) in *Ppm1d*^T/+^ driven AML compared to WT. **C** GSEA shows relevant pathways upregulated in oncogenic transformation. Oncogenic signatures (MSigDB) significantly enriched (FDR < 0.25) in *Ppm1d*^T/+^ driven AML compared to WT. **D** Expression of selected genes in AML patients (*n* = 151) vs. healthy controls (*n* = 407) from TNMplot database. **E** Kaplan–Meier survival analysis of AML patients from TCGA dataset based on IGF2BP3 expression.

To further characterize the *Ppm1d*^T/+^ leukemic cells, we performed gene expression analysis of the BM c-Kit^+^ cells isolated from recipients that were transplanted with the primary leukemias (Fig. 4A). Principal component analysis revealed that while *Ppm1d*^T/+^ AMLs were heterogeneous, the WT leukemias shared a highly homogenous transcriptome, reflecting that they originated from the sole WT primary leukemia (Supplimentary Fig. 3B). Overall, we identified 2444 differentially expressed genes (Log2FC > 1, *p*-adjusted value < 0.05) including 1645 genes upregulated in *Ppm1d*^T/+^ AML (Fig. 4B). GSEA further confirmed that the transcriptome of *Ppm1d*^T/+^ AML was enriched for gene signatures typical for AML, MDS, and other cancers (Fig. 4C). Interestingly, the most significantly upregulated genes in *Ppm1d*^T/+^ AML (*Zbed3, Runx3, Marcks, Extl3, Pcbp4, Igf2bp3, Plch1*, and *Gdf6*) were previously associated with AML and cancer progression [52–58]. We compared the expression of these genes in AML patients versus healthy samples using the TNM database [42], and noted that *PLCH1*, *ZBED3*, *IGF2BP3*, *INSM1*, and *SLC17A8* were elevated in leukemic patients in comparison to healthy samples (Fig. 4D). Out of these, increased expression of PLCH1 was previously associated with imatinib-resistant CML [59]. Further, the RNA-binding protein *IGF2BP3* has previously been implicated in the progression of AML [53, 54, 60] and other cancers [61, 62]. Remarkably, patient stratification based on *IGF2BP3* showed that high levels of this insulin like growth factor 2 mRNA binding protein correlated with worse survival (Fig. 4E). Altogether, these results suggest that the transcriptomic profile of *Ppm1d*^T/+^ AMLs correlates with a more aggressive disease in human myeloid malignancies, and that our *Ppm1d*^T/+^ murine model exhibits certain similarities to human AMLs.

### Inhibition of PPM1D impairs the colony forming potential of *Ppm1d*^T/+^ cells

**Fig. 5:**
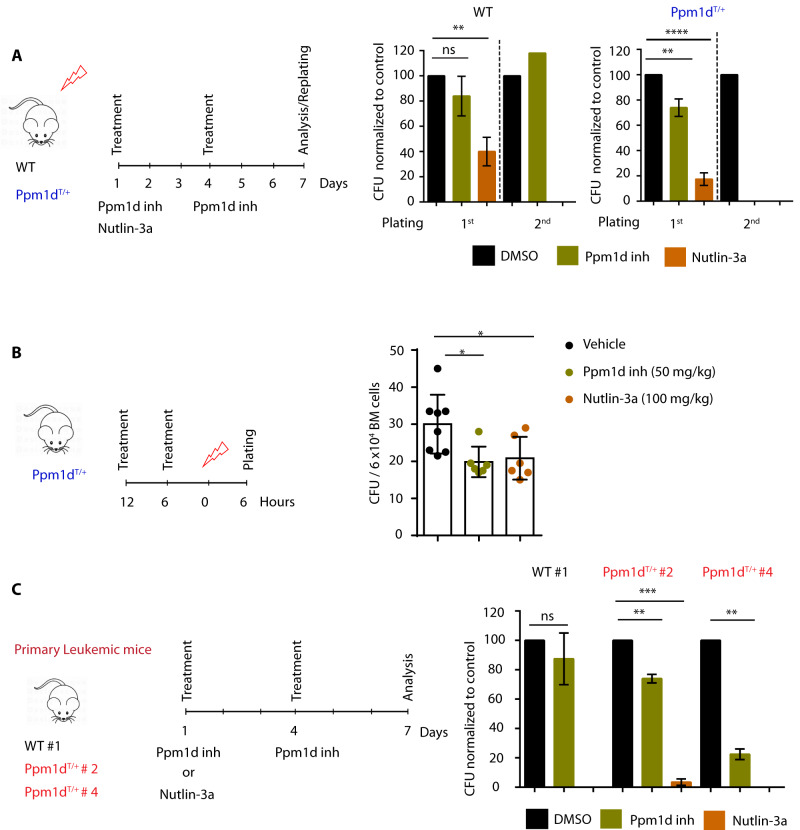
Impaired growth of *Ppm1d*^*T*/+^ cells upon Ppm1d inhibition. **A** Schematic representation of the experimental design. WT and *Ppm1d*^T/+^ mice were exposed to 3 Gy sub-lethal irradiation. Six hours after irradiation a total of 6 × 10^4^ BM cells were plated per well with the indicated doses of PPM1D inhibitor (GSK2830371) or Nutlin-3a (MDM2 inhibitor). The Y-axis indicates the percentage of colony forming units (CFU) normalized to DMSO control. Each group contained at least 3 animals from 2 independent experiments. Data represent mean ± s.d. Statistical significance was determined by 2-tailed Student’s *t*-tests (****p < 0.0001, **p < 0.01, ns: not significant). **B** Schematic representation of the experimental design. *Ppm1d*^T/+^ mice were gavaged with PPM1D inhibitor (GSK2830371), Nutlin-3a (MDM2 inhibitor), or vehicle control 12 and 6 h prior to sub-lethal irradiation. Colony-forming assay in MethoCult M3434 was performed 6 h post irradiation. The y-axis indicates the total number of CFU per 6 × 10^4^ BM cells. At least 6 mice were used per group. Data represent mean ± s.d. Statistical significance was determined by 2-tailed Student’s *t*-tests (*p < 0.05). **C** Schematic representation of the experimental design. Colony culture assays of BM cells isolated from WT irradiated leukemic mice (WT#1) and *Ppm1d*^T/+^ irradiated leukemic mice (*Ppm1d*^T/+^ #2 and #4) in the presence of PPM1D inhibitor (GSK2830371), Nutlin-3a (MDM2 inhibitor), or vehicle control (DMSO). Colonies were enumerated on day 7 of culture. Data represent mean ± s.d. Statistical significance was determined by using 2-tailed Student’s *t*-tests (***p < 0.001, **p < 0.01, ns: not significant).

We hypothesized that the proliferation of *Ppm1d*^T/+^ BM cells upon irradiation is enabled by increased activity of PPM1D that partially suppresses p53 pathway. To test this, we exposed the WT and *Ppm1d*^T/+^ mice to ionizing radiation, isolated BM cells 6 h later and subjected them to colony culture assays in the presence of Ppm1d inhibitor GSK2830371, MDM2 inhibitor Nutlin-3a or vehicle control (Fig. 5A). While we were able to maintain the *Ppm1d*^T/+^ BM cells in culture for up to 2 rounds of plating in the presence of vehicle control, the colony forming ability was severely impaired when Ppm1d was inhibited (Fig. 5A, right panel). Similarly, colony formation of *Ppm1d*^T/+^ BM cells was suppressed by upregulation of p53 pathway by Nutlin-3a (Fig. 5A, right panel). Further, the effects on WT cells were only significant when Nutlin-3a was added to the cultures and not PPM1D inhibitor (Fig. 5A, left panel). Altogether, these assays indicate that PPM1D inhibitor and Nutlin-3 suppressed cell growth of *Ppm1d*^T/+^ BM cells isolated from mice exposed to ionizing radiation and that the Ppm1d inhibitor effects are restricted to cells expressing the truncated and stabilized form of Ppm1d.

Next, we investigated whether Ppm1d inhibition could be used to prevent the irradiation-induced expansion in vivo. To this end, *Ppm1d*^T/+^ mice were pre-treated with vehicle control, Ppm1d inhibitor, and Nutlin-3a for 12 and 6 h prior to irradiation. Mice were sacrificed 6 h after irradiation and BM cells were placed in culture (Fig. 5B). Interestingly, we observed that Ppm1d inhibition, as well as Nutlin-3a treatment, significantly reduced colony formation in comparison to vehicle control (Fig. 5B). This suggests that stimulation of p53 function either by inhibition of PPM1D or by Nutlin-3a treatment prior to irradiation, could suppress the proliferation advantage of HSPCs carrying truncated PPM1D and thus prevent cell transformation.

Finally, we investigated whether PPM1D inhibition was sufficient to prevent the growth of primary leukemic cells in culture. Upon isolation of leukemic WT and leukemic *Ppm1d*^T/+^ BM cells, cultures were established in the presence of PPM1D inhibitor, Nutlin-3a, or vehicle control (Fig. 5C). Remarkably, while PPM1D inhibition had no effect on the growth of the leukemic cells carrying the WT *Ppm1d*, it significantly reduced the expansion of leukemic cells derived from *Ppm1d*^T/+^ mice (Fig. 5C). Altogether, these results indicate that inhibition of PPM1D suppresses colony-forming potential of AML carrying the truncated PPM1D. Ultimately, our data suggest that truncated PPM1D enhances abnormal growth of t-AML, and inhibition of PPM1D may represent a promising treatment strategy for a fraction of therapy-induced leukemias.

## Discussion

Gain-of-function mutations in *PPM1D* leading to expression of a stable PPM1D protein have been implicated in solid tumors (including breast [26, 63], colon cancer [27], and glioma [64, 65]) and in age-related or therapy-induced clonal hematopoiesis [28, 29]. In addition, PPM1D mutations in a CH model have recently been shown to promote inflammation and non-ischemic heart failure in mice [34]. Here, we used a *Ppm1d*^T/+^ transgenic mouse model to investigate how truncated PPM1D affects the hematopoietic system and leukemogenesis. We demonstrated that expression of the truncated PPM1D is associated with the decline of HSPC numbers, BM cellularity, and shorter lifespan of mice. Despite the phenotypic analysis showing reduced hematopoietic output and HSCs numbers, extreme limiting dilution transplantation assay revealed enhanced repopulating ability of *Ppm1d*^T/+^ HSCs. As the enhanced proliferation and activation of HSC results in the gradual decline of HSC functionality and numbers [66], our results suggest that the aberrant proliferative advantage of *Ppm1d*^T/+^ HSCs might be responsible for reduced production of mature blood cells in adult *Ppm1d*^T/+^ mice. On the contrary to our results, Yura et al did not observe any differences in leukocyte counts 4 weeks after BM reconstitution with HSPC transduced with lentivirus expressing Cas9 targeting the exon 6 of *Ppm1d* [34]. It is plausible that this HSC phenotype could not be detected in a short-term analysis, where repopulation is mostly driven by progenitors and where only ~60% of HSCs contained truncated PPM1D. In addition, in their study expression of truncated PPM1D is restricted to hematopoietic cells, while in our model, truncated PPM1D is ubiquitously expressed, and thus, we cannot exclude that expression of PPM1D in the BM niche further enhances the hematopoietic phenotype. Nevertheless, exploring this possibility is beyond the scope of our study and should be investigated in the future. The subsequent transcriptomic analysis of WT and *Ppm1d*^T/+^ HSC did not show any differentially expressed genes under basal conditions. We speculate that PPM1D-mediated suppression of p53 pathway can occur only during DNA damage response that would, at a time, affect only an undetectable fraction of HSCs [67], but that could potentially result in gradual depletion of HSC pool over longer periods [68]. Indeed, unbiased GSEA analysis consistently revealed the inclination of *Ppm1d*^T/+^ towards cell cycle deregulation as well as upregulation of the mTOR pathway and reduced NOTCH signaling. Since both pathways were reported to affect HSC self-renewal via modulating symmetry of cell division [44, 45], we quantified the division types of HSCs doublets based on the Numb levels. We found a significant increase in cellular differentiation and a decrease of symmetric self-renewal divisions in *Ppm1d*^T/+^ HSCs in comparison to WT HSCs. As Numb was reported to inhibit NOTCH activity [48, 49], this result is aligned with our GSEA results and provides additional explanation for the reduced HSC numbers in the *Ppm1d*^T/+^ mice. Nevertheless, whether the other hematopoietic populations are also reduced solely as a result of altered HSC self-renewal, or whether other factors contribute to the phenotype, remains to be addressed. In this study, we did not observe significant changes in *Numb* expression in the population of *Ppm1d*^T/+^ HSCs, indicating that Numb is likely regulated at protein rather than transcription level. As Numb phosphorylation has been associated to asymmetric cell division, we searched for potential upstream regulators and noted diminished levels of Camk1 and negative enrichment of genes involved in the Ca2 + /calmodulin-dependent protein kinase pathway in *Ppm1d*^T/+^ HSCs. These observations allow us to speculate that Numb phosphorylation might be reduced in *Ppm1d*^T/+^ HSCs, and consequently affect the type of HSC division [50, 51, 69]. Nevertheless, the precise molecular mechanism underlying the aberrant distribution of Numb in *Ppm1d*^T/+^ HSCs remains to be addressed.

Previous reports identified high incidence of the truncating PPM1D mutations in patients with therapy-induced AML [28, 29] and established the role of PPM1D as a driver in CH, but whether these mutations can contribute to malignant transformation per se remained elusive. Our experiments demonstrate that *Ppm1d*^T/+^ mice frequently develop AML upon sequential exposure to low doses of ionizing radiation. As PPM1D activity can efficiently suppress the checkpoint function of p53 [13–17, 27], our observations suggest that truncated PPM1D enables continuous proliferation of *Ppm1d*^T/+^ HSPCs upon therapy-induced DNA damage that may promote genome instability and cell transformation. According to our gene expression analysis, radiation induced *Ppm1d*^T/+^ AML in mice shares transcriptomic features associated with poor prognosis and reduced survival in AML patients [53, 54, 60]. Thus, our data from transgenic mice are consistent with a proposed model in which truncating PPM1D mutations represent a risk factor for the development of therapy-induced AML [28, 29].

Finally, we report that the proliferative advantage of irradiated BM cells isolated from *Ppm1d*^T/+^ mice exposed to ionizing radiation is lost after inhibition of PPM1D activity or by stimulation of p53 function by inhibition of MDM2. Importantly, the effect of PPM1D inhibitor was restricted only to cells expressing the truncated and stabilized form of PPM1D. We propose that inhibition of PPM1D allows cells to establish the checkpoint after radiotherapy preventing genome instability and cell transformation. Ultimately, our data allow us to speculate that prophylactic inhibition of PPM1D may prevent individuals exhibiting CH from acquiring additional driving mutations and thus protect them from the development of hematological malignancies. Similarly, leukemias developed in *Ppm1d*^T/+^ mice were sensitive to inhibition of PPM1D in culture indicating that PPM1D may represent a pharmacological target in therapy-induced leukemias. Nevertheless, this observation needs to be validated in vivo once new PPM1D inhibitors with better solubility and favorable pharmacokinetic properties become available [70, 71]. Our research justifies the need for large population studies allowing a precise evaluation of the cancer risk in carriers of truncating PPM1D mutations as well as the development of new PPM1D inhibitors.

### Supplementary information

Supplementary information

## Data Availability

The complete data set from RNAseq analysis was deposited in ArrayExpress database (https://www.ebi.ac.uk/biostudies/arrayexpress) under accession numbers E-MTAB-13056 and E-MTAB-13090.
