# Supplementary material for: Ppm1d truncating mutations promote the development of genotoxic stress-induced AML
Source: Leukemia. 2023 Sep 14;37(11):2209–20. doi: 10.1038/s41375-023-02030-8 (PMC10624630; doi:10.1038/s41375-023-02030-8)
Supplement: Supplementary file 1 — Supplementary information [file 41375_2023_2030_MOESM1_ESM.pdf]

Ppm1d truncating mutations promote the development of genotoxic stress-induced AML

Monika Burocziova[1, 2], Petr Danek[2], Anna Oravetzova[1], Zuzana Chalupova[1], Meritxell Alberich-Jorda[2,3,#], and Libor Macurek[1,#]

Supplementary information

Supplementary text. **Supplementary Methods**

Supplementary Figure 1. **Generation and characterization of *Ppm1d*$^{T/+}$ mice.**

Supplementary Figure 2. **Transcriptional and functional analysis of *Ppm1d*$^{T/+}$ HSCs.**

Supplementary Figure 3. **Effects of irradiation on *Ppm1d*$^{T/+}$ mice.**

Methods

Phenotypic analysis. The murine hematopoietic tissues were analyzed using the following antibodies: Gr1 PE (RB6-8C5), CD11b APC (M1/70), Ly6G FITC (1A8), c-kit PE (2B8), Sca-1 APC (D7), CD34 FITC (RAM 34), FcγRII/III Pe-Cy7 (93), CD48 FITC (HM48-1), CD150 Pe-Cy7 (TC15-12F12.2), anti-mouse Lineage Cocktail Pacific Blue (including CD3 (17A2); Gr1 (RB6-8C5); CD11b (M1/70); CD45R/B220 (RA3-6B2); TER-119 (Ter-119)). Antibodies were purchased from Biolegend.

Whole BM transplantation. Transplanted mice were analyzed using Ly5.1 FITC (A20), Ly5.2 PE-Cy7 (104), Gr1 APC (RB6-8C5), CD11b APC (M1/70), CD45/B220 APC, CD45/B220 PE (RA3-6B2), and CD3e PE (145-2C11). Primary leukemic donor (Ly5.2+) BM was isolated from WT and Ppm1d$^{T/+}$ animals. 1×10^5 of donor cells were intravenously transplanted in sub-lethally irradiated recipients (Ly5.1$^+$). PB was analyzed weekly after transplantation. Cells were stained with anti-Ly5.1 and Ly5.2 antibodies (to distinguish donor-derived cells), lineage-specific antibodies (CD11b FITC, Gr1 PE, B220, and CD3) and c-kit$^+$ APC myeloblasts. Alternatively, for RNA isolation the enriched leukemic c-Kit$^+$ cells were isolated using MACS separator. Antibodies were purchased from Biolegend.

Extreme limiting dilution transplantation assays (ELDA). C57BL/6NCrl mice (CD45.1$^+$) at age of 10 – 12 weeks were used as recipients and were lethally irradiated (6 Gy) prior to transplantation. Donor cells were isolated from WT and PPM1D$^{T/+}$ (Ly5.2$^+$) murine BM. HSCs, defined as LKS CD48$^-$, CD150$^+$ were sorted and intravenously transplanted at three different doses (5, 10, and 20 cells) along with 5×10^5 BM cells as a support (Ly5.1$^+$). BM and blood of recipients were analyzed 16 weeks after

transplantation. Cells were stained with anti-Ly5.1 and Ly5.2 antibodies to distinguish donor-derived cells from the support cells, and with lineage-specific antibodies CD11b, Gr1, B220, and CD3 to assess the reconstitution of myeloid cells, B-cells, and T-cells, respectively. A recipient mouse was defined as positive when engraftment of donor cells was ≥ 0.1% and presented at least two lineages reconstituted. The frequency of HSCs was calculated with ELDA online software using Poisson statistics and the method of maximum likelihood to the proportion of negative recipients in a limiting dilution setting[1].

RNA sequencing and analysis. RNA sequencing and analysis of HSCs (HSCs, defined as Lin^- $c\text{-}Kit^+$, $Sca\text{-}1^+$, $CD48^-$, $CD150^+$ cells) sorted from 12-week-old WT and $Ppm1D^{T/+}$ mice (n=4) using Influx instrument (BD Biosciences) was performed. RNA was extracted with the RNAeasy Micro Kit (Qiagen) and cDNA was synthesized using the SMARTer Stranded Total RNA-Seq Kit v2 Pico (Takara Bio) according to the manufacturer's instructions. Alternatively, RNA was isolated from c-Kit+ cells isolated from the leukemic animals using KAPA mRNA HyperPrep Kit (Kapa Biosystems). Sequencing was performed by NextSeq 550 system (Illumina) using NextSeq 500/550 High Output Kit v2 (75 cycles). Sequences were mapped to GRCm39 reference genome by STAR v2.7.9 software. The raw counts for each transcript were calculated using featureCounts software from Rsubread package for R software[2]. Differential expression and normalized counts were determined by DESeq2 package in R. Genes with expression change higher than log2FoldCHange > 1 and p-adjusted value < 0.05 were considered as significantly up- or down-regulated. Gene Set Enrichment Analysis was performed as described[3]. All genes were pre-ranked based on the statistical significance of gene expression change -log10(p-adjusted value) for leukemic samples, -log10 (p-value) for HSC and their enrichments in the gene sets available at MSigDB were analyzed. Gene expression patterns in AML patients versus healthy individuals were obtained from TNMplot database[4].

Western blotting analysis. BM was isolated from WT and $Ppm1d^{T/+}$ mice and fraction of c-Kit+ cells was enriched using MACS separator. Extracted cells were lysed (65 mM Tris pH 6.8, 10 % glycerol, 1 % SDS, 0.5 % DTT). Protein lysates were separated using reducing SDS-PAGE (10% acrylamide gel), transferred to nitrocellulose membrane, and stained with anti PPM1D Rabbit (clone D4F7, Cell Signaling, #11901) and Importin-β (Santa Cruz, sc-137016) antibodies.

Colony culture assays. Murine myeloid colony culture assays were performed using Methocult GF M3434 (Stemcell Technologies). For re-plating assays $1×10^4$ whole BM cells were initially plated, cells were harvested after 7 days, and $1×10^4$ cells were re-plated. For irradiated and leukemic mice, $6x10^4$ cells were plated and harvested after 7 days, and the same number was re-plated. Colonies and cell counts were scored after 7 days of *in vitro* culture. Where indicated, cells were incubated in the presence of GSK2830371 (1 μM, MedChem Express, hereafter referred to as PPM1D inhibitor[5,6]) or

MDM2 inhibitor nutlin-3a (10 μM, MedChem Express)[7]. Counting was performed by investigators blinded to the genotype.

Immunohistochemistry. Spleen was harvested and placed in a 4% formaldehyde diluted in PBS overnight and then transferred into 70% ethanol. Paraffin-embedded sections were cut 2,5 μm thick and deparaffinized, cleared, and rehydrated in graded ethanol concentrations. To examine the morphological changes in the spleen, standard hematoxylin and eosin staining protocol was followed. The Histopathology report was examined by a pathologist at Czech Centre for Phenogenomics of the Institute of Molecular Genetics.

Immunofluorescence. Sorted HSC cells were cultured in StemSpam medium for 40 hours in the presence mIL-3 (10 ng/ml), hIL-6 (20 ng/ml), mSCF (100 ng/ml), mTPO (50 ng/ml), mFlt3 (100 ng/ml). After 24 h, cytochalasin B (1.5 μg/ml) was added for 16 hours. Cells were sedimented on poly-lysine-coated glass slides using cytospin, dried, fixed with 4 % paraformaldehyde, permeabilized with 0.3% Triton™ X-100 and stained with Numb antibody (1:500, Cell Signaling) according to the manufactures' recommendation. All samples were counterstained with 4,6-diamidino-2-phenylindole (DAPI). Images were acquired using IX81 microscope (Olympus) equipped with ScanR imaging platform using a 40x/1.3 NA objective with oil immersion. Quantification of GFP signal intensity was performed using ImageJ 1.54b. Higher Numb protein expression in one of two daughter HSCs indicates an asymmetric division, detection of high Numb expression in both daughter cells shows symmetric differentiation divisions, while sustained low levels of Numb in both daughter cells marks symmetric HSC self-renewal divisions. Asymmetric division was defined as at least two-fold difference in the intensity of Numb between paired daughter cells. At least 60 cell pairs were counted in each sample.

Statistical analysis. Statistical significance for indicated data sets was determined using unpaired 2-tailed Student t-test and p values <0.05 were considered as statistically significant. Scatter dot plots depict mean with error bars representing standard deviation (s.d.). Survival analysis was performed using the Kaplan-Meier method and log-rank Mantel-Cox test was used to assess statistical significance of survival differences between experimental groups. The number of mice was chosen to ensure 90% power with a 5% error based on the observed standard deviation. The number of animals in each experiment is indicated in the figure legend.

Suppl. Fig. 1

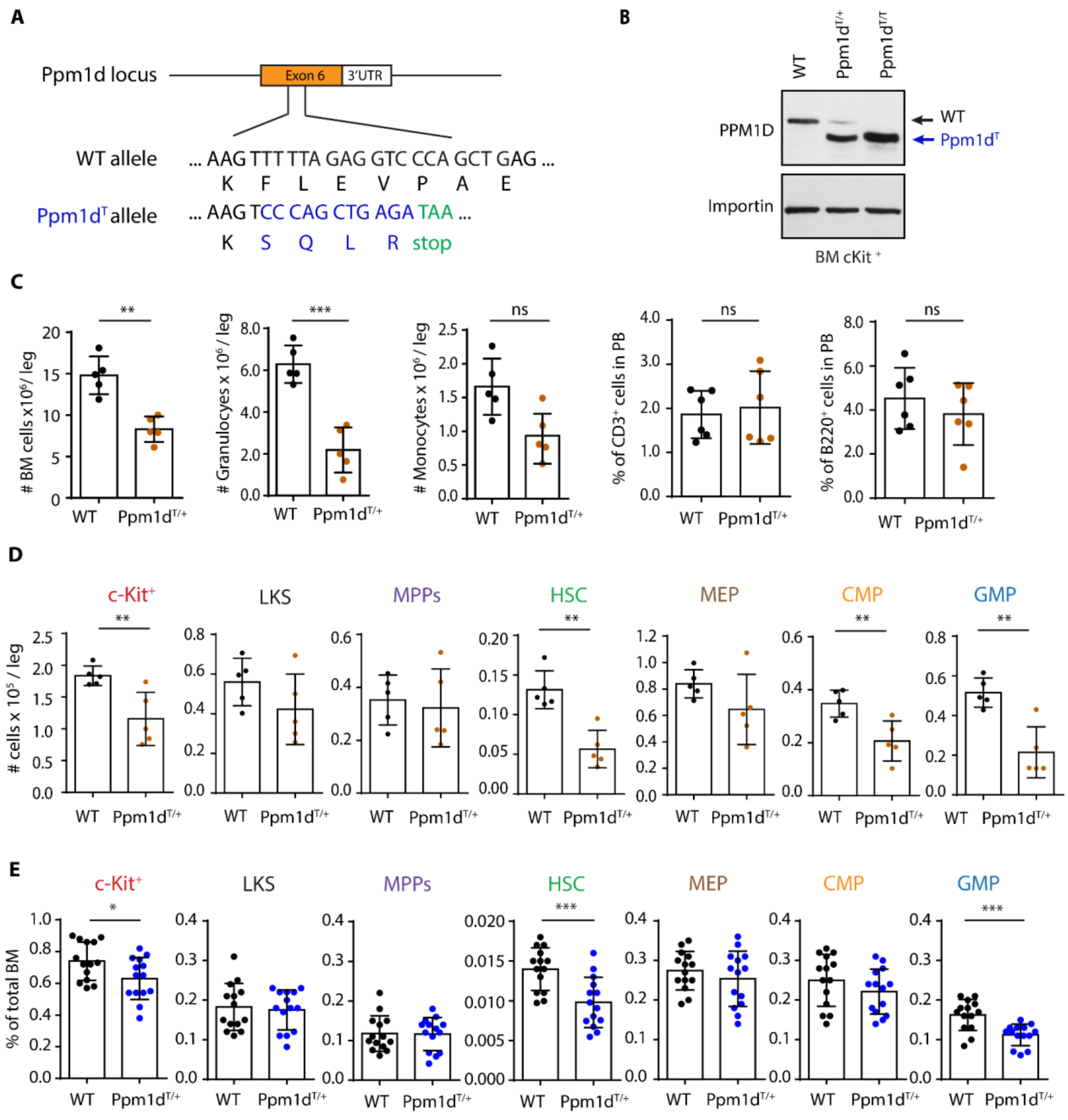

Supplementary Fig. 1. Generation and characterization of *Ppm1d*$^{T/+}$ mice.

(S1A) Schematic representation of TALEN-mediated editing of the *Ppm1d* locus. DNA and protein sequences of the wild type *Ppm1d*$^{+/+}$ (WT) and *Ppm1d*$^{T/+}$ alleles are shown. The frameshift mutation that resulted in the truncated protein with 4 new amino acids is indicated in blue.

(S1B) Immunoblot analysis of truncated PPM1D in BM enriched c-Kit+ cells. The black arrow indicates the positions of WT and the blue arrow indicates truncated PPM1D. Importin was used as a loading control.

(S1C) Absolute number of BM cells, monocytes ($CD11b^+Ly6C^+$), and granulocytes ($CD11b^+Ly6G^+$) per leg and percentage of CD3+ (T cells) and B220+ (B cells) in peripheral blood in 58-week-old animals. Each symbol represents one mouse. At least 5 animals were used per group. Data represent mean ± s.d. Statistical significance was determined by 2-tailed Student's t-tests (***$p < 0.001$, **$p < 0.01$, ns: not significant).

(S1D) Absolute number of distinct HSPC populations in WT and *Ppm1d*$^{T/+}$ BM isolated from 58-week-old mice. c-Kit$^+$ (Lin$^-$ c-Kit$^+$ Sca-1$^-$), LKS (Lin$^-$ c-Kit$^+$ Sca-1$^+$), MPP (Lin$^-$ c-Kit$^+$ Sca-1$^+$ CD48$^+$ CD150$^-$), HSC (Lin$^-$ c-kit$^+$ Sca-1$^+$ CD48$^-$ CD150$^+$), MEP (Lin$^-$ c-kit$^+$ Sca-1$^-$ CD34$^-$ FcgRII/III$^-$), CMP (Lin$^-$ c-Kit$^+$ Sca-1$^-$ CD34$^+$ FcgRII/III^{lo}), and GMP (Lin$^-$ c-Kit$^+$ Sca-1$^-$ CD34$^+$ FcgRII/III^{hi}). Each symbol represents one mouse. Data represent mean ± s.d. Statistical significance was determined by 2-tailed Student's t-tests (**$p < 0.01$).

(S1E) Percentages of distinct HSPC populations (as indicated in panel E) in BM isolated from WT and *Ppm1d*$^{T/+}$ 12-week-old mice. Each symbol represents one mouse. Data represent mean ± s.d. Statistical significance was determined by 2-tailed Student's t-tests (***$p < 0.001$, *$p < 0.05$).

Suppl. Fig. 2

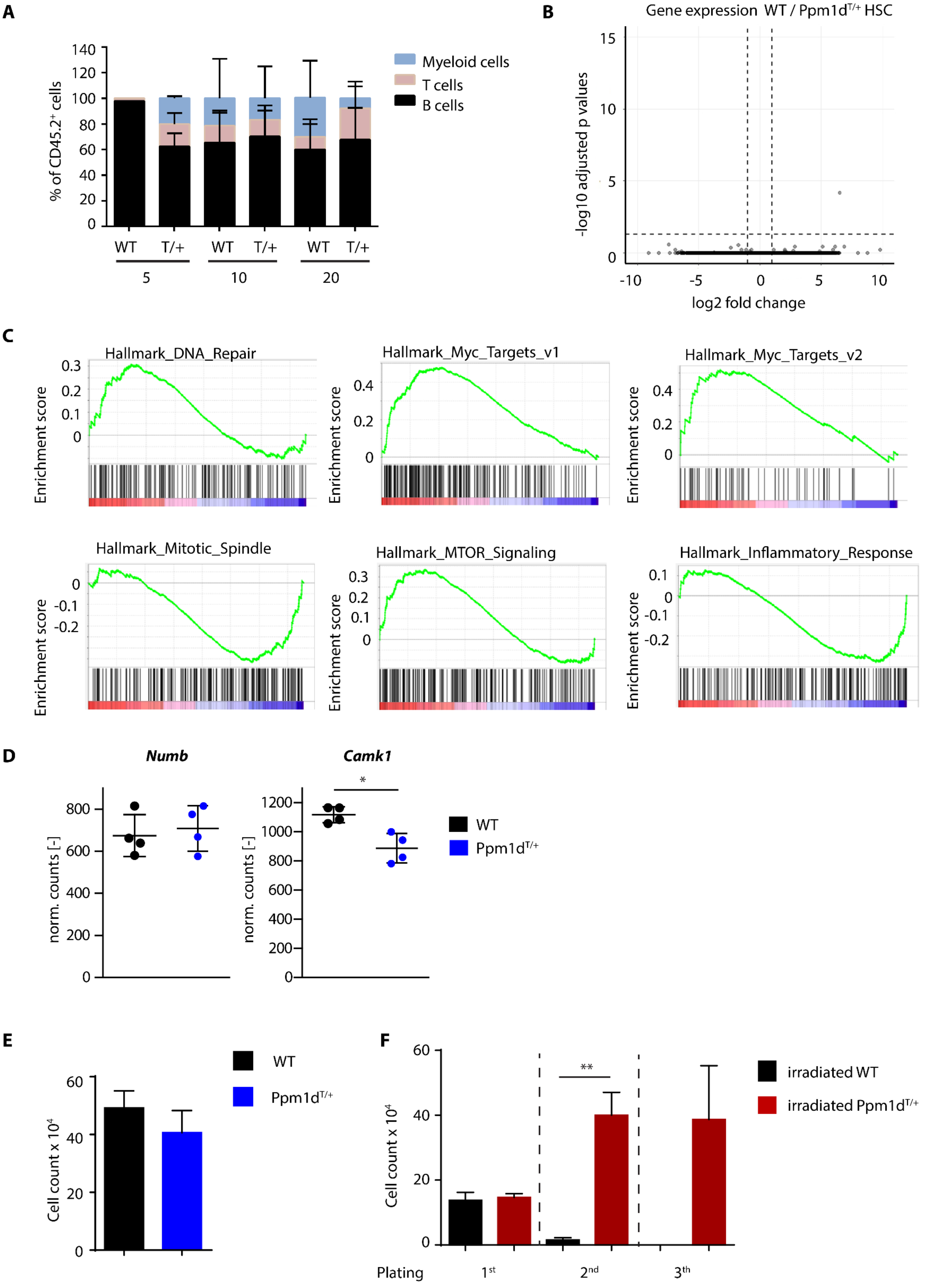
A
% of CD45.2+ cells
Myeloid cells
T cells
B cells
WT T/+ WT T/+ WT T/+
5 10 20
B
Gene expression WT / Ppm1dT/+ HSC
-log10 adjusted p values
log2 fold change
C
Hallmark_DNA_Repair
Hallmark_Myc_Targets_v1
Hallmark_Myc_Targets_v2
Hallmark_Mitotic_Spindle
Hallmark_MTOR_Signaling
Hallmark_Inflammatory_Response
Enrichment score
D
Numb
Camk1
norm. counts [-]
WT
Ppm1dT/+
E
Cell count x 10^4
WT
Ppm1dT/+
F
Cell count x 10^4
irradiated WT
irradiated Ppm1dT/+
Plating
1st
2nd
3th

Supplementary Fig. 2. Transcriptional and functional analysis of Ppm1dT/+ HSCs.

(S2A) Tri-lineage reconstitution upon transplantation of WT and Ppm1dT/+ (T/+) HSCs. The x-axis indicates the dose of donor cells (5, 10, or 20 HSCs). The y-axis indicates the percentage of cells defined as Gr1+ CD11b+ myeloid cells (blue), B220+ B-cells (black), and CD3+ T-cells (pink).

(S2B) Volcano plot showing differences in gene expression between Ppm1dT/+ and WT HSCs.

(S2C) Representative enrichment plots from GSEA analysis of six individual hallmarks showing deregulated pathways in Ppm1dT/+ HSC.

(S2D) Expression values of *Numb* and *Camk1* genes in WT (black symbols) and Ppm1dT/+ HSCs (blue symbols) showed as number of normalized counts. Bars indicate median +/- SD. Statistical significance was determined by DEseq2 software (* p<0.05).

(S2E) Number of cells in colony culture assays. 1x104 BM cells isolated from WT (black) and Ppm1dT/+ (blue) mice were plated per well. At least three mice were used per group in two separate experiments. Data represent mean ± s.d. Statistical significance was determined by 2-tailed Student's t-tests (ns: not significant).

(S2F) Number of cells in colony culture replating assays. Mice were exposed to 3 Gy sub-lethal IR and 6 h after exposure a total of isolated 6x104 BM cells was plated per well. Colonies were counted and replated on day 7. At least three mice were used per group in two independent experiments. Data represent mean ± s.d. Statistical significance determined by 2-tailed Student's t-tests (**p < 0.01).

Suppl. Fig. 3

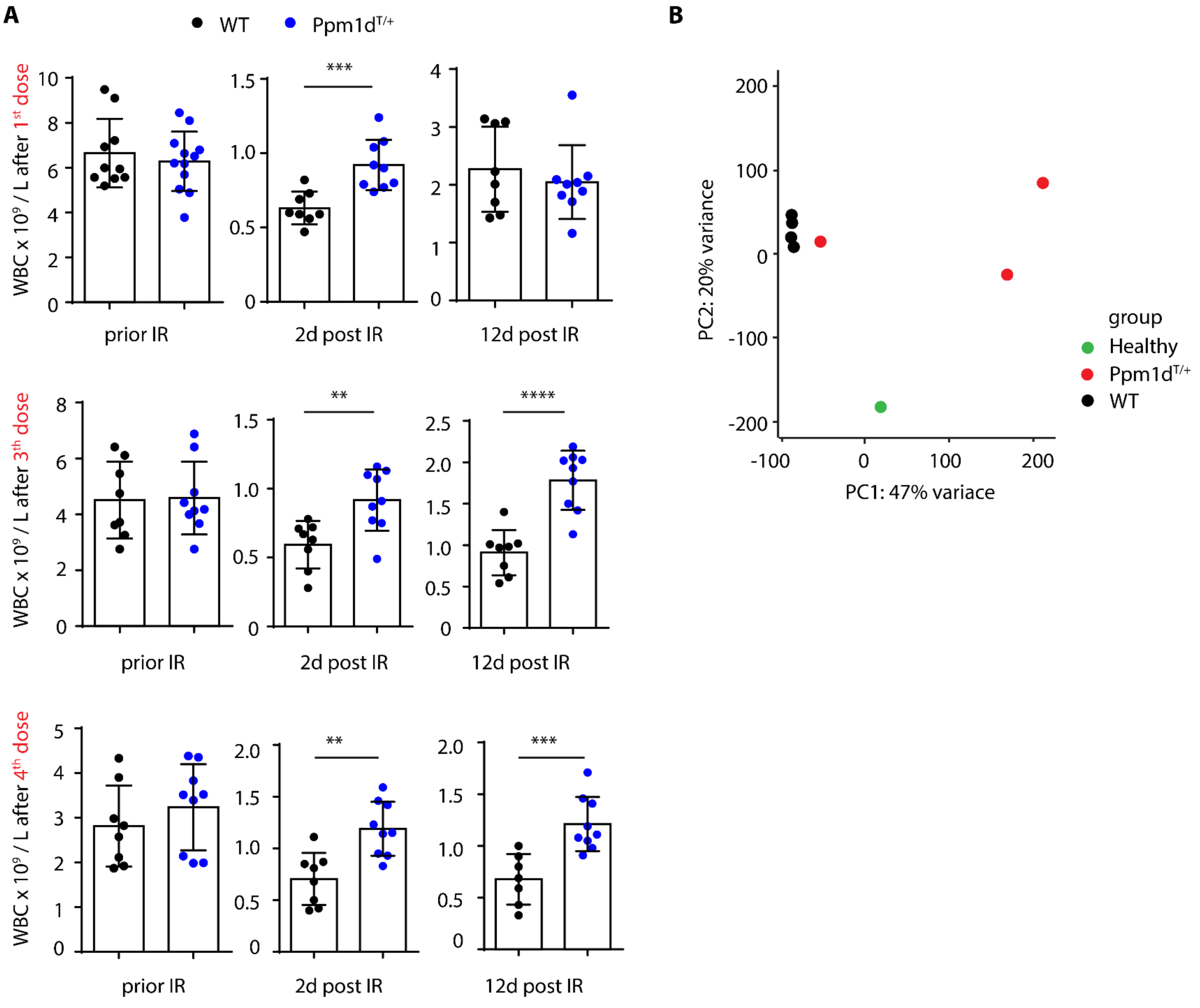

Supplementary Fig. 3. Effects of irradiation on *Ppm1d*$^{T/+}$ mice.

(S3A) Number of white blood cells (WBC) per liter in PB of 12-week old WT and *Ppm1d*$^{T/+}$ mice upon irradiation based on Auto Hematology Analyzer. PB was analyzed prior to IR, 2 and 12 days after exposure to ionizing radiation (3 Gy). Each symbol represents one mouse. Data represent mean ± s.d. Statistical significance was determined by 2-tailed Student's t-tests (****p < 0.0001, ***p < 0.001, **p < 0.01).

(S3B) Principal component (PC) analysis of four leukemic WT samples (WT#1; black symbols), three *Ppm1d*$^{T/+}$ samples (red symbols), and one healthy WT sample (green).

1. Hu Y, Smyth GK. ELDA: extreme limiting dilution analysis for comparing depleted and enriched populations in stem cell and other assays. *J Immunol Methods*. 2009;347(1-2):70-78.
2. Liao Y, Smyth GK, Shi W. featureCounts: an efficient general purpose program for assigning sequence reads to genomic features. *Bioinformatics*. 2014;30(7):923-930.
3. Subramanian A, Tamayo P, Mootha VK, et al. Gene set enrichment analysis: a knowledge-based approach for interpreting genome-wide expression profiles. *Proc Natl Acad Sci U S A*. 2005;102(43):15545-15550.
4. Bartha Á, Győrffy B. TNMplot.com: A Web Tool for the Comparison of Gene Expression in Normal, Tumor and Metastatic Tissues. *International Journal of Molecular Sciences*. 2021;22(5):2622.
5. Gilmartin AG, Faitg TH, Richter M, et al. Allosteric Wip1 phosphatase inhibition through flap-subdomain interaction. *Nat Chem Biol*. 2014;10(3):181-187.
6. Miller PG, Sathappa M, Moroco JA. Allosteric inhibition of PPM1D serine/threonine phosphatase via an altered conformational state. *Nat Commun*. 2022;13(1):3778.
7. Vassilev LT, Vu BT, Graves B, et al. In Vivo Activation of the p53 Pathway by Small-Molecule Antagonists of MDM2. *Science*. 2004;303(5659):844-848.
